# Latitudinal variation in biophysical characteristics of avian eggshells to cope with differential effects of solar radiation

**DOI:** 10.1002/ece3.4335

**Published:** 2018-07-17

**Authors:** Jesús Gómez, Cristina Ramo, Martin Stevens, Gustavo Liñán‐Cembrano, Miguel A. Rendón, Jolyon T. Troscianko, Juan A. Amat

**Affiliations:** ^1^ Departamento de Ecología de Humedales Estación Biológica de Doñana (EBD‐CSIC) Sevilla Spain; ^2^ Centre for Ecology and Conservation College of Life and Environmental Sciences University of Exeter Penryn UK; ^3^ Instituto de Microelectrónica de Sevilla (IMSE‐CNM CSIC/Universidad de Sevilla) Sevilla Spain

**Keywords:** biogeographical pattern, biophysical mechanisms, *Charadrius alexandrinus*, egg coloration, egg size, latitudinal gradient, UV protection

## Abstract

Solar radiation is an important driver of animal coloration, not only because of the effects of coloration on body temperature but also because coloration may protect from the deleterious effects of UV radiation. Indeed, dark coloration may protect from UV, but may increase the risk of overheating. In addition, the effect of coloration on thermoregulation should change with egg size, as smaller eggs have higher surface‐volume ratios and greater convective coefficients than larger eggs, so that small eggs can dissipate heat quickly. We tested whether the reflectance of eggshells, egg spottiness, and egg size of the ground‐nesting Kentish plover *Charadrius alexandrinus* is affected by maximum ambient temperature and solar radiation at breeding sites. We measured reflectance, both in the UV and human visible spectrum, spottiness, and egg size in photographs from a museum collection of plover eggshells. Eggshells of lower reflectance (darker) were found at higher latitudes. However, in southern localities where solar radiation is very high, eggshells are also of dark coloration. Eggshell coloration had no significant relationship with ambient temperature. Spotiness was site‐specific. Small eggs tended to be light‐colored. Thermal constraints may drive the observed spatial variation in eggshell coloration, which may be lighter in lower latitudes to diminish the risk of overheating as a result of higher levels of solar radiation. However, in southern localities with very high levels of UV radiation, eggshells are of dark coloration likely to protect embryos from more intense UV radiation. Egg size exhibited variation in relation to coloration, likely through the effect of surface area‐to‐volume ratios on overheating and cooling rates of eggs. Therefore, differential effects of solar radiation on functions of coloration and size of eggshells may shape latitudinal variations in egg appearance in the Kentish plover.

## 1 INTRODUCTION

One of the most thoroughly studied topics of animal coloration is that of melanism, the occurrence of individuals that are darker in pigmentation. Several hypotheses have been advanced to explain the existence of melanism, the most prominent including links to camouflage and solar radiation, such as thermoregulation and protection from ultraviolet (UV) radiation (see Clusella‐Trullas, van Wyk, & Spotila, 2007). These different functions of pigmentation should place considerable selection pressure on animal appearance, which in turn should also be influenced by environmental factors such as solar radiation and habitat.

The steady‐state temperature that an organism reaches in the absence of metabolic heating and evaporative cooling is referred to as operative temperature, which depends on absorbed radiation, air temperature, and wind speed (Bakken, 1992). Given that coloration affects the amount of energy absorbed or reflected at different wavelengths, the operative temperature of an organism may be affected by its color. Under the thermoregulation hypothesis, melanism is advantageous in cold climates, and lighter individuals are expected to occur in hotter areas given the lower absorptance by light colors (Bishop et al., 2016). Consistent with this hypothesis, a geographical variation in the degree of melanism has been found in various taxa of ectotherms, with darker individuals being found at higher latitudes and lighter ones at lower latitudes (Alho et al., 2010; Brakefield, 1984; Moreno Azócar et al., 2015; Rapoport, 1969). The UV resistance hypothesis posits that dark colors reduce the transmission of UV light through body layers (Bastide, Yassin, Johanning, & Pool, 2014; Thompson, 1955). Therefore, changes in color to cope with temperature and UV radiation are likely when differences in body temperature and UV protection affect fitness (Clusella‐Trullas, Terblanche, Blackburn, & Chown, 2008; Umbers, Herbestein, & Madin, 2013).

The same principles that are applied to ectotherms regarding the relationship between the radiative environment and coloration might be applied to eggshells. Therefore, as in ectotherms, changes in coloration with solar radiation should be expected in avian eggshells. The eggs may remain exposed to environmental conditions during absences of adults from nests, which may be critical for embryos of species nesting at ground level in exposed sites, as unattended eggs receiving solar radiation may overheat in a very few minutes (Amat, Gómez, Liñán‐Cembrano, Rendón, & Ramo, 2017; Amat & Masero, 2007; Grant, 1982; Wilson‐Aggarwal, Troscianko, Stevens, & Spottiswoode, 2016). Avoiding overheating is important because high body temperatures denature proteins. Likely because of this there are some biophysical mechanisms with which birds may counteract the negative effects of high temperature on embryos’ overheating. One of such mechanisms is eggshell color, as lighter eggs overheat less quickly than darker eggs when exposed to direct solar radiation (Gómez et al., 2016; Lahti & Ardia, 2016; Montevecchi, 1976). However, the eggshell spotting typical of many species makes the eggs darker (Gómez et al., 2016; Troscianko, Wilson‐Aggarwal, Stevens, & Spottiswoode, 2016), so that the degree of spottiness may affect the rates of egg overheating. At least during the first moments after egg exposure to direct solar radiation, it may be expected that spots overheat quicker than the eggshell background (see Wacker, McAllan, Körtner, & Geiser, 2016), although after longer exposure, the eggs may reach equilibrium temperatures across their surfaces.

Eggshell color may also affect embryo viability through the probability of UV transmittance throughout the eggshell (Veterány, Hluchý, & Veterányová, 2004). UV‐B is strongly mutagenic, and most mutations are harmful (e.g., de Gruij & Forbes, 1995). Because the eggs contain the DNA, harmful mutations could be inherited by the next generation (e.g., Flenley, 2011). Darker colors reduce light transmittance through eggshells, thus protecting the embryo from UV radiation (Abram et al., 2015; Brulez, Pike, & Reynolds, 2015; Gaudreau, Abram, & Brodeur, 2017; Lahti & Ardia, 2016; Maurer et al., 2015; Shafey, Ghannam, Al‐Batshan, & Al‐Ayed, 2004). Because the absorptance by darker colors is higher than that of lighter colors, this may result in a trade‐off between the risk of egg overheating and the risk of UV radiation on embryos if ambient temperature and solar radiation are positively related (Lahti & Ardia, 2016): Darker eggshells would protect the embryo from UV radiation, but in turn would increase the risk of overheating. However, the thermoregulation and UV protection hypotheses are not necessarily mutually exclusive, for instance when the relationship between solar radiation and temperature is nonlinear, as it may occur in tropical mountains where radiation is high but temperature low.

The effect of coloration on thermoregulation should change with egg size, so that there would be a positive relationship between egg size and dark coloration (Gates, 1980), a prediction supported by theoretical models and empirical evidence (Clusella‐Trullas et al., 2008; Schweiger & Beierkuhnlein, 2016). Therefore, another biophysical mechanism with which birds may counteract the negative effects of high temperature on embryos’ overheating is egg size, as smaller eggs have higher surface‐volume ratios and greater convective coefficients than larger eggs, so that small eggs can dissipate heat quickly (e. g., Porter & Gates, 1969).

**Figure 1:**
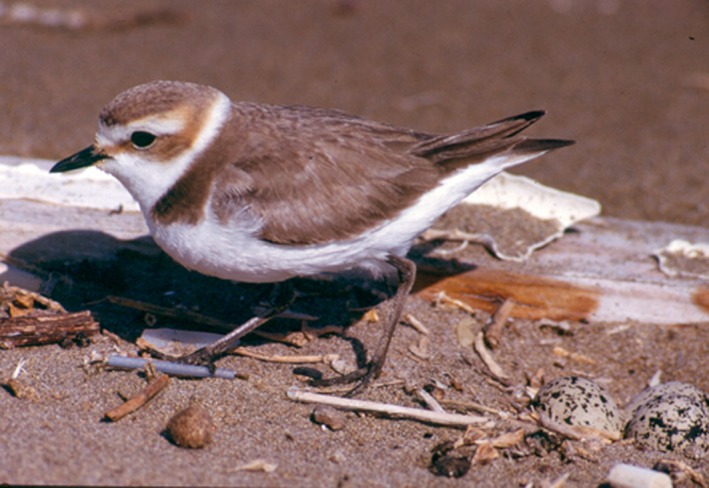
Female Kentish plover (*Charadrius alexandrinus*) beside its nest (photo credit: Xavier Ferrer)

Given the above considerations, depending on the environment, the importance of selective drivers on eggshell coloration may vary spatially, leading to complex interactions among factors that affect variations in color patterns (Ahlgren, Yang, Hansson, & Brönmark, 2013; Bastide et al., 2014; Lindstedt, Linström, & Mappes, 2008), as well as in egg size, to cope with solar radiation in different environments. The resolution of the above trade‐offs would depend on which effects are more limiting on embryos’ viability. In this study, we tested whether the reflectance of Kentish plover *Charadrius alexandrinus* eggshells, both in the human visible (VIS) and UV spectrum, is affected by maximum ambient temperature (which may affect egg temperatures through thermal convection) and solar radiation (including total maximum radiation incident on the surface of Earth, which may affect egg temperatures through thermal radiation) across a large geographical range. Kentish plovers are small shorebirds that usually nest in exposed sites that receive direct solar radiation (Figure 1). We also tested the relationship between both coloration and spotting patterns of eggshells and egg size. We expected that eggshells with higher reflectance (i.e., those of lighter colors) and less spotting should be found in sites where solar radiation is higher. However, if UV radiation is very high, the impact of UV radiation on embryo viability may be stronger than the risk of egg overheating, in which case eggshells with lower reflectance and more spotting should be expected in sites with very high solar radiation levels, even if the risk of egg overheating is high (see Bastide et al., 2014). Lastly, we tested whether there are variations in egg size in relation to eggshell color, so that lighter eggs should be smaller, given the thermal advantages of such eggs in hotter environments.

## 2 METHODS

### 2.1 Study species and photography

The Kentish plover is a shorebird widely distributed across the Palearctic. It nests at ground level in exposed sites. Lack of nest cover allows a quick detection of approaching predators by incubating birds (Amat & Masero, 2004a), but eggs overheat to temperatures that may be lethal for embryos in <1 min when left unattended by adults and receiving direct solar radiation (Amat & Masero, 2007; Amat et al., 2017; Grant, 1982). The absences of shorebirds from nests may last 3–10 min, but there are cases lasting >4 hr (Pedler, Weston, & Bennett, 2016), suggesting that there may be selective pressure on eggs to have pigmentation that would protect them against adverse effects of solar radiation.

**Figure 2:**
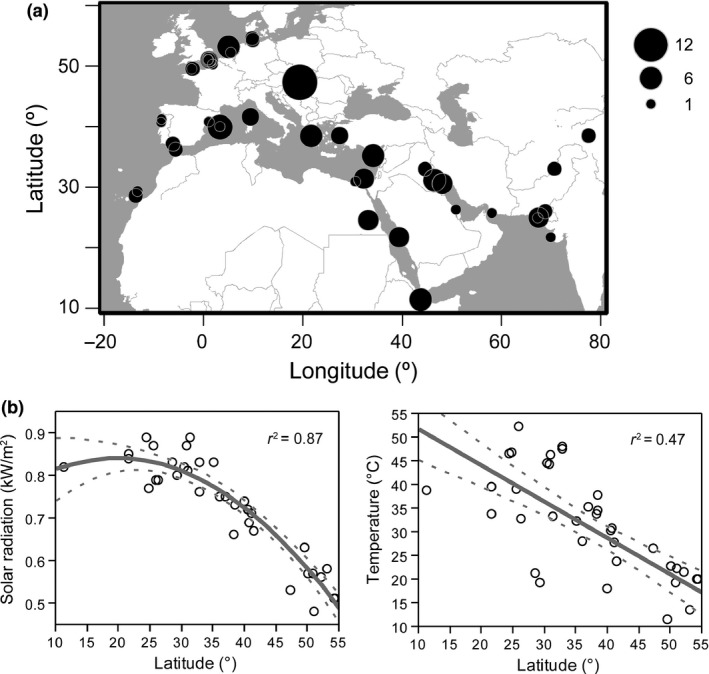
(a) Map showing the spatial distribution of collection sites for eggs of Kentish plover. Symbol size is proportional to sample size. (b) Relationships between latitude and both solar radiation and temperature in the localities where the eggs were collected

For this study, we used the Kentish plover eggshells in the collection of the Natural History Museum at Tring (United Kingdom). We noted the date and locality of collection of every clutch. Using Google^TM^ Earth, we obtained the coordinates of those localities. The eggs had been collected between 1,858 and 1,972, across a geographical range encompassing 11.35°N–54.52°N and 13.86°W–77.25°E (Figure 2a).

Eggshells were photographed following the protocols of Stevens, Párraga, Cuthill, Partridge, and Troscianko (2007), Troscianko and Stevens (2015), and Gómez and Liñán‐Cembrano (2017). Only one eggshell per clutch (n = 110) was photographed using standardized lighting conditions provided by a UV lamp (EYE Color ARC MT70D, Iwasaki Electric Co., Ltd., Tokyo, Japan) diffused with a silver photographic umbrella, using a Nikkon D7000 camera with a 105 mm Micro‐Nikkor lens that transmits UV (see Troscianko et al., 2016). The camera had undergone a quartz conversion to allow the sensor to detect UV light. Using a Baader UV‐IR blocking filter (Baader Planetarium, Mammendorf, Germany), we took photographs in the human visible spectrum (400–700 nm, which reflects light in the RGB), and with a Baader UV‐pass IR blocking filter, we could take images in the UV spectrum (approximately 315–400 nm). The camera was mounted on a camera stand, and photographs were taken, with a shutter cable, at f/4 in RAW format. We placed beside the eggs a Spectralon grey standard (40% UV‐visible, Labsphere, Congleton, UK) that reflects light in the UV and human visible spectra. A metric scale was included in all photographs.

### 2.2 Image analysis

All images were linearized with respect to radiance using the tool SpotEgg (Gómez & Liñán‐Cembrano, 2017). Values from the Spectralon grey standard were employed for normalization to standardize images with regard to the illuminating light and convert the data to percent reflectance (Gómez & Liñán‐Cembrano, 2017; Stevens et al., 2007; Troscianko & Stevens, 2015). Once the reflectance images were generated, the next step was to manually draw closed polygonal lines defining the regions of interest (RoIs), where spottiness and color analyses were executed. Finally, we analyzed color and spottiness. For this, SpotEgg employs an image‐processing algorithm to segment the spots from the background in each of the RoIs on an image. The spot detection routine produced detailed.CSV reports with information about reflectance (in the red, green, and blue camera's color bands) for spots (S) and eggshell background (B), as well as the size of each detected spot, the number of spots, and total spottiness (percentage of the area of eggshell covered by spots).

For the UV images, the sensitivity of the camera system (filter + lens + image sensor) in the UV is significantly lower than for the visible wavelengths, requiring longer exposure times, such that images signal‐to‐noise ratio is degraded. These two facts may pose difficulties to detect the spots in the UV images. Consequently, we opted for using the images of spots detected in the visible image as a mask for evaluating reflectance of both spots and eggshell background in the UV band. Although we tried to keep the camera position when the filters were changed, it was inevitable that the UV and visible images were in practice taken from slightly different positions, so that both images were not coincident. Hence, using spot information from the visible image as a mask to measure reflectance in the UV images required applying a space‐variant geometrical transformation to the mask image to ensure proper matching. We implemented a plugin for SpotEgg that inferred the spatial transformation between the visible and UV images from a set of at least 13 corresponding points (manually marked as feature matching did not work due to the low‐quality UV images). Once the control pairs of points had been selected, SpotEgg employed the Local Weighted Mean method proposed by Goshtasby (1998)(especially well suited when the distortion varies locally) to find the geometrical transformation between the UV and visible images. This transformation was applied to the black and white image with the location of the spots that were produced for the visible image. The result was another black and white image corresponding to spots that were correctly aligned with their corresponding UV image. This UV‐spot image was then used as a mask to obtain the reflectance of both eggshell spots and background in the UV band.

As measures of color, we used the mean values of the three camera bands (red, green and blue) in the VIS, and the red band in the UV. In addition, using SpotEgg (Gómez & Liñán‐Cembrano, 2017), we also estimated egg volume (mm^3^) and surface (mm^2^), which resulted from integrating RoIs as a revolving surface shape generator.

### 2.3 Environmental variables

We obtained data on the monthly average amount of the total radiation incident on a horizontal surface at the surface of the Earth at noon (average of 3 hr at the time closest to the local solar noon), as well as maximum daily Earth's surface temperature from NASA (https://eosweb.larc.nasa.gov/cgi-bin/sse/grid.cgi?email=skip@larc.nasa.gov). The meteorological data were on a scale of 1‐degree longitude by 1‐degree latitude, covering the entire range from which eggs were obtained, and were averaged on a monthly basis over a 22‐year period (July 1983–January 2005) for the corresponding quadrat of every locality. We only used data for the months during which the Kentish plover is breeding at every locality (laying dates obtained from Wiersma, Kirwan, & Boesman, 2016). There is a latitudinal gradient in radiation and temperature across the localities at which the eggs were collected (Figure 2b).

### 2.4 Statistical procedures

We performed a principal components analysis (PCA) from a correlation matrix on eggshell color and spot patterning to obtain a smaller set of uncorrelated components that represents most of the information in the original variables. Before PCA, log‐transformation linearized the relationships between egg color and spot variables.

Generalized additive mixed models (GAMMs) were applied to estimate spatial variation in egg color and patterning (Wood & Augustin, 2002). In contrast to GAMs, GAMMs permit spatio‐temporal correlation within blocks using random effects. In order to model spatial patterns, we included the latitude of nests as a smoother. Furthermore, the spatial model included the effect of the year of collection as linear covariate to test for temporal changes in egg coloration and patterning during long‐term storage (Cassey, Maurer, Duval, Ewen, & Hauber, 2010; Navarro & Lahti, 2014). Finally, the collection site names were considered as a random effect, resulting in a semiparametric mixed model.

The influence of the environment (temperature and solar radiation) on eggshell color and spot patterns was also analyzed using GAMMs, with collection site name as a random effect. Environmental models included year as a linear covariate if this variable was previously chosen in the spatio‐temporal model. Furthermore, we tested the linear effect of egg size (surface area‐to‐volume ratio) on egg coloration (Gates, 1980). The small‐sample‐size corrected version of Akaike information criterion (AICc; Burnham, Anderson, & Huyvaert, 2011) was used to compare competing models (Zuur, Ieno, Walker, Savaliev, & Smith, 2009). Models with ≤2 AIC of the top model are considered as competitive. However, uninformative parameters with models ≤2 ΔAICc (i.e., do not explain enough variation) were interpreted as having no effect on the response (Arnold, 2010). Using a full fixed effect model, we assessed the influence of the random component using AICc based on REML estimators. The optimal fixed effects structure was also selected using AICc, but based on ML estimators. We restricted the GAMMs to a maximum of five knots to prevent over‐fitting. Then, we checked if polynomial or linear models fitted better to data than GAMM. For the parametric models, the relative explanatory power of fixed (insolation and temperature) and random (site identity) effects was determined using conditional and marginal *R* ^2^s for Generalized mixed‐effect models (Nakagawa & Schielzeth, 2013). We measured for concurvity (the generalized additive model analogue to collinearity) between covariates (Hastie & Tibshirani, 1990), which may result in inaccurate estimates of the GAM functions (Ramsay, Burnett, & Krewski, 2003). The level of concurvity varies between 0, no concurvity, and 1, total lack of identifiability (Wood, 2017). In this study, pairwise values of observed concurvity between explanatory variables, both in spatio‐temporal and environmental models, ranged from low to moderately high (0.27–0.69). Residuals from the environmental models for eggshell color and spot patterns did not present a large‐scale latitudinal trend (PC1: edf (equivalent degrees of freedom) = 2.66, *p* = 0.167; PC2: edf = 1.94, *p* = 0.374) nor spatial patterns, as determined by semivariance analysis and Mantel's test (results not shown). GAMMs were conducted with R software version 3.3.3 (R Core Team, 2017) using the packages *mgcv* 1.8–17 (Wood, 2006, 2017) and *MuMIN* 1.15.6 (Barton, 2016).

## 3 RESULTS

### 3.1 Eggshell reflectance and spot patterns

**Figure 3:**
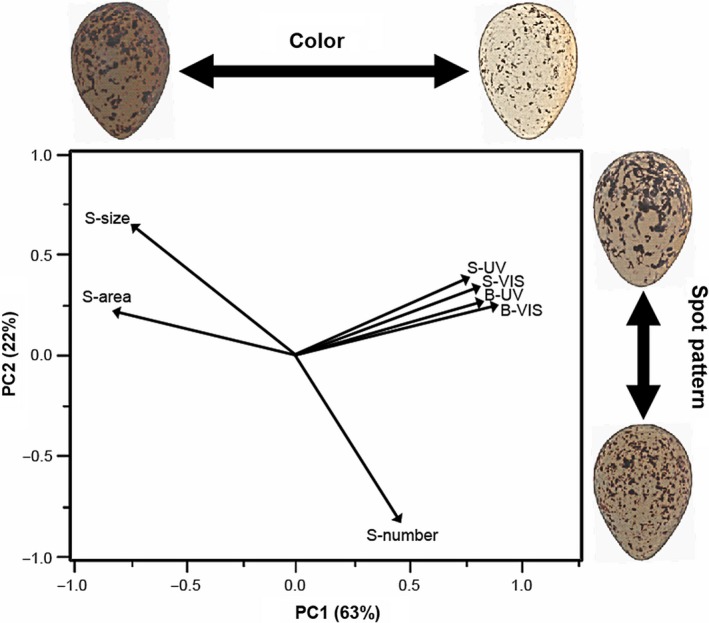
Biplot of the two‐first principal components for the variables used to describe both eggshell (S, spots, B, background) color in the human visible (VIS) and ultraviolet (UV) spectrum, and spot pattern (size, area, and number) in the Kentish plover

Eggshell reflectance and spot pattern variables were intercorrelated, except spot number and reflectances (Table 1). The two‐first principal components accounted for 85% of the variance of egg color and spot pattern, so that we retained them for further consideration (Figure 3). The first principal component (PC1) accounted for 63% of the variance and was related to eggshell reflectance, with negative values indicating eggs with large spots and darker backgrounds and spots, as well as eggshells and spots that reflected less in the UV (eigenvectors: spot number = 0.229, spot size = −0.360, spottiness = −0.400, background VIS = 0.428, spot VIS = 0.390, background UV = 0.406, and spot UV = 0.398). The second principal component (PC2) accounted for 22% of the variance and related to patterning; positive values of PC2 were related to decreasing spot number but increasing spot size (spot number = −0.655, spot size = 0.523, spotiness = 0.181, background VIS = 0.218, spot VIS = 0.295, background UV = 0.220, spot UV = 0.286).

**Table 1:** Pearson's correlation coefficients between variables describing eggshell reflectance, for both background (B) and spots (S), in the visible (VIS) and ultraviolet (UV) spectrum, and spot patterning (number, size, and area) in the Kentish plover

	B‐VIS	B‐UV	S‐UV	S‐number	S‐size	S‐area
S‐VIS	0.79***	0.63***	0.84***	0.12 ns	−0.39***	−0.59***
B‐VIS		0.90***	0.74***	0.22*	−0.49***	−0.66***
B‐UV			0.77***	0.21*	−0.45***	−0.60***
S‐UV				0.17 ns	−0.40***	−0.55***
S‐number					−0.88***	−0.43***
S‐size						0.81***

*Notes*

ns, nonsignificant.

*p < 0.05; *^*^ *p* < 0.01; *^**^ *p* < 0.001.

### 3.2 Spatio‐temporal model

The AICc values for the models including latitude and year as fixed effects indicate that adding the site as a random effect significantly improved model performance, both for eggshell reflectance (∆AICc = 3.68) and spot patterning (∆AICc = 5.82).

**Figure 4:**
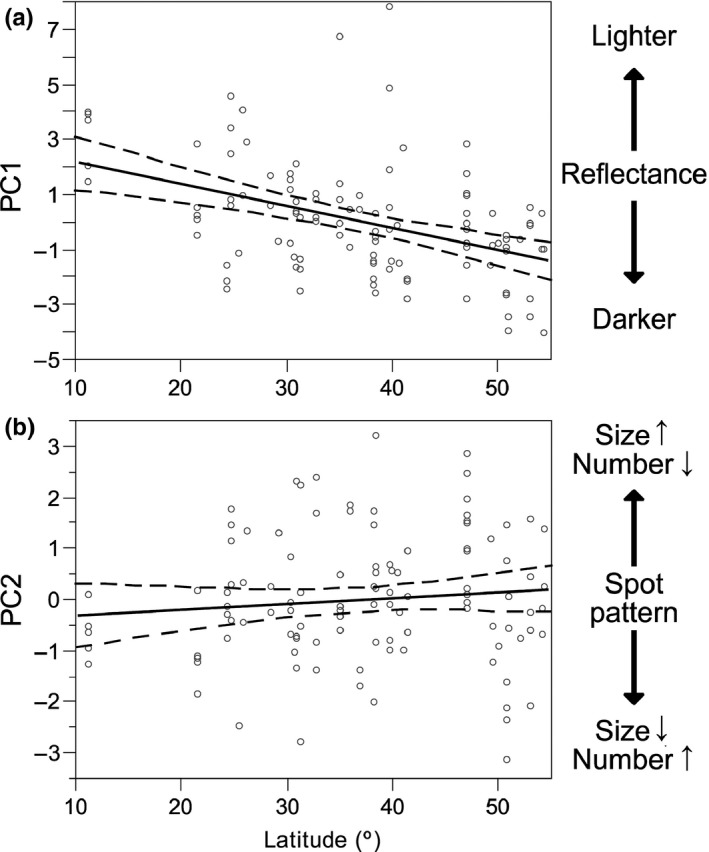
(a) Partial plots (95% CI) of GAMMs for the relationships between latitude and eggshell reflectance (PC1). (b) Spot patterning (PC2). Only the significant environmental variables were included in each model

The eggshell reflectance (PC1) showed a spatial and temporal variation (Table 2). Despite the GAMM that included year as a linear predictor and latitude as a smoother was highly supported (AICc = 450.59), including latitude as a linear term significantly improved model performance (AICc = 448.35). Eggshells tended to be lighter and with more reflectance in the UV toward lower latitudes (Figure 4a; *β* = −0.052 ± [*SE*] 0.022, *p* = 0.019). Once spatial structure was accounted for, the year of collection was related to eggshell color, with older eggs being darker than those collected later (*β* = 0.027 ± 0.010, *p* = 0.010). The linear model had $R_{m}^{2}$  = 0.24 due to spatio‐temporal pattern, and a *R* ^2^ = 0.15 due to random effects.

**Table 2:** Results of the spatio‐temporal GAMMs fitted to eggshell reflectance (PC1) and spot patterning (PC2)

Dependent variable	Fixed effects	*df*	AICc	∆AICc	*w*_i_	$R_{m}^{2}$	$R_{c}^{2}$
PC1	Year + s (latitude)	6	450.59	2.24	0.206	–	–
	Year + latitude	5	448.35	0.00	0.632	0.24	0.39
	Latitude	4	453.16	4.81	0.057	0.18	0.39
	Year	4	452.00	3.61	0.104	0.17	0.37
	Null model	3	464.12	16.12	0.000	0.00	0.37
PC2	Year + s (latitude)	6	361.12	4.24	0.061	–	–
	Year + latitude	5	358.88	2.00	0.167	0.03	0.27
	Latitude	4	358.68	1.80	0.168	0.01	0.27
	Year	4	358.11	1.22	0.223	0.01	0.27
	Null model	3	356.89	0.00	0.381	0.00	0.27

*Note*

Parametric coefficients for latitude were estimated when the degrees of freedom for the spline estimates (s) equals 1. Collection site was entered as an intercept‐only random effect in both models. The difference between the lowest AIC and the AICc score of each model (ΔAICc), the Akaike weight (*w*_i_), the variance explained by the fixed factors ($R_{m}^{2}$ ), and the variance explained by the entire model ($R_{c}^{2}$ ) are presented.

Spot pattern (PC2) did not show significant spatial or temporal patterns (Figure 4b). A null model including only random intercepts for site provided the best fit (Table 2).

### 3.3 Environmental models

For eggshell reflectance (PC1), the lower values of AICc were achieved when the model included year, egg surface‐to‐volume ratio, and insolation as explanatory variables (Table 3). Temperature had no significant relationship with PC1. Surface area‐to‐volume ratio of the eggs was also positively related to PC1 (*β* = 69.850 ± 23.191, *p* = 0.004), and thus, larger surface area‐to‐volume ratios (smaller eggs) tended to be associated with lighter‐colored eggshells.

**Table 3:** Results of the environmental GAMMs fitted to eggshell reflectance (PC1) and spot patterning (PC2)

Model	*df*	AICc	∆AICc	*w*_i_	$R_{m}^{2}$	$R_{c}^{2}$
PC1						
Year + sv + s (insolation) + s (temperature)	9	451.83	22.85	0.000	–	–
Year + s (insolation) + s (temperature)	8	452.36	23. 38	0.000	–	–
Sv + s (insolation) + s (temperature)	8	449.81	20.83	0.000	–	–
Year + sv + s (insolation)	7	447.14	18.16	0.000	–	–
Year + sv + s (temperature)	7	447.81	18.83	0.000	–	–
Year + sv + insolation^3^	8	442.02	13.04	0.001	0.34	0.47
Year + sv + insolation^4^	9	428.98	0.00	0.998	0.45	0.48
Null model	3	464.12	35.14	0.000	0.00	0.37
PC2						
s (insolation) + s (temperature)	7	363.76	6.88	0.014	–	–
s (insolation)	5	360.06	3.18	0.089	–	–
s (temperature)	5	361.23	4.35	0.049	–	–
Insolation	4	357.87	0. 98	0.266	0.02	0.26
Temperature	4	359.04	2. 15	0.148	0.00	0.27
Null model	3	356.89	0.00	0.434	0.00	0.27

*Note*

Parametric linear effects were applied for surface/volume (sv) of eggs and collection year. Smoothing spline functions (s) were used both for temperature and solar radiation, then linear and polynomial (cubic and quartic) models were also fitted. Collection site was entered as a random effect in both models.

ΔAICc, the difference between the lowest AICc and the AICc score of each model; *w*_i_, Akaike weight; $R_{m}^{2}$ , marginal *R* ^2^; $R_{c}^{2}$ , conditional *R* ^2^.

**Figure 5:**
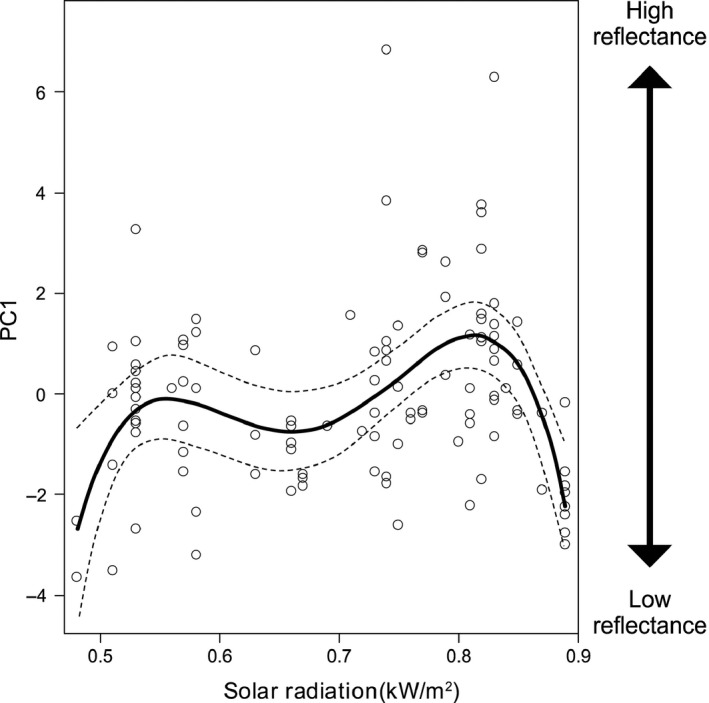
Partial plots (95% CI) of a four‐order polynomial model for the relationships between solar radiation and eggshell color (PC1). Only the significant environmental variables were included in each model. Rug plot represents the spread of the data

Eggshell reflectance responded in a nonlinear fashion to solar radiation (Figure 5). The nonparametric smoothing function of solar radiation was significant (e.d.f. = 3.715, *p* = 0.002). When the smooth term was replaced by a fourth‐order polynomial term the model fit improved (Table 3). Eggshell color became lighter from low values of solar radiation until reaching 0.55 kW/m^2^ (Figure 5). Thereafter, eggshell color varied slightly until radiation at ground level was near 0.7 kW/m^2^, and then became even lighter until values of 0.8 kW/m^2^, but with higher radiation levels, eggshells became darker again. Marginal *R* ^2^ indicates that the fixed terms accounted for most of the explained variance of PC1 ($R_{m}^{2}$  = 0.45) relative to random effect (0.03).

For spotting pattern (PC2), two models were within 2 AICc units of each other and competed for best model for the variation in spot pattern (Table 3). The model with the lower AICc value for PC2 was the null model (*w*_i_ = 0.415). The second best‐fit mixed model contained the linear effect of solar radiation but it was not significant (*β* = −1.289 ± 1.216, *p* = 0.295), had a lower Akaike weight (*w*_i_ = 0.266), and only explained 2% of the variation. Therefore, we considered radiation as a uninformative variable on spotting pattern. These results suggest that the spatial variation of eggshell patterning was rather site‐specific ($R_{c}^{2}$  = 0.27).

## 4 DISCUSSION

The study of geographical variations in phenotypic traits is useful in the understanding of the selective agents that shape the phenotypic variation (Roulin & Randin, 2016). Our results indicate that eggshell reflectance in the Kentish plover across a latitudinal gradient, both in the human visible and UV spectrum, is primarily determined by spatial variations in solar radiation, and that this may also affect egg size in a way that has not been appreciated in previous studies (see Williams, 1994). Although some studies have shown that larger eggs are laid in colder environments (Azevedo, French, & Partridge, 1996; Liefting, Weerenbeck, van Dooremalen, & Ellers, 2010), we show for the first time a relationship between egg color and size, as smaller eggs (i.e., those with higher surface‐volume ratio) tend to be lighter than larger eggs. Relative to darker eggs, light eggs overheat more slowly when exposed to direct solar radiation (Gómez et al., 2016), and smaller eggs in hot environments may cool down relatively quickly compared with larger eggs because heat dissipation increases with surface/volume ratio (Porter & Gates, 1969). All this indicates that information of egg size in relation to eggshell coloration may be important to forecast environmental change sensitivity of ground‐nesting birds. In contrast to this, birds nesting in cup nests above ground level use nest‐building techniques as an adaptive external phenotype, which can mitigate some of these physical effects in hot environments (Crossman, Rohwer, & Martin, 2011; Heenan, Goodman, & White, 2015).

An alternative explanation for the observed association between egg size and pigmentation may be due to lighter eggs being smaller simply because small eggs and limited pigment may be two correlated characteristics of eggs laid by female birds that are in poor condition. That is, individual females that cannot invest as much in egg size (producing large eggs is costly) are also limited in their ability to invest in pigment production (if pigment is costly) (e.g., Siefferman, Navara, & Hill, 2006). However, this could imply that there are geographical gradients in the body condition of females, which seems unlikely.

As solar radiation is mainly affected by latitudinal variations, it may be concluded that the pattern in eggshell coloration is latitudinal, with eggshells of darker color and lower UV reflectance found in northern latitudes, although this is conditioned by high solar radiation levels (see below). Thermal constraints may partly drive the observed spatial variation in eggshell coloration, which may be lighter to diminish the risk of overheating (Gómez et al., 2016; Lahti & Ardia, 2016). In ground‐nesting birds, darker eggs can sometimes have better camouflage than lighter eggs (Gómez et al., 2016; Troscianko et al., 2016), which suggests that ground‐nesting birds may face trade‐offs between the level of egg camouflage and dealing with high levels of solar radiation (Gómez et al., 2016; Wilson‐Aggarwal et al., 2016). However, in sites where solar radiation is very high (>0.8 kW/m^2^), lighter colors may not be advantageous because of the higher transmittance of light‐colored eggshells, assuming no variation in eggshell thickness, as this increases the risk of UV radiation reaching the embryo (Abram et al., 2015; Brulez et al., 2015; Gaudreau et al., 2017; Lahti & Ardia, 2016; Maurer et al., 2015). Such high UV radiation levels are found at low latitudes, and this may be a reason for the occurrence of eggs of dark coloration at such latitudes in spite of high levels of solar radiation. Studies with *Drosophila* flies have shown latitudinal clines in pigmentation, with darker phenotypes being found at higher latitudes. However, the relationship is reversed at southern latitudes near the Equator, with UV radiation being the main factor responsible for this reversed latitudinal pattern (Bastide et al., 2014), similar to our results.

We expected that ambient temperatures could have affected eggshell coloration. However, there was no significant relationship between these variables, likely because incubating plovers may control egg temperature using several behavioral strategies (Amat & Masero, 2004b; Vincze et al., 2017), and thus, the risks of egg overheating becomes relevant only after the eggs remain exposed to direct solar radiation (i.e., when incubating birds depart from nests).

The Kentish plover, as other polygamous plovers, exhibits low genetic structure and weak isolation‐by‐distance (D'Urban Jackson et al., 2017; Küpper et al., 2012; Oyler‐McCance, St. John, Kysela, & Knopf, 2008; Vidal, Hernández, Luis, & Domínguez, 2015). Despite gene flow, there may be geographic phenotypic divergence in response to changes in local conditions (Bech et al., 2016; Ritcher‐Boix, Teplitsky, Rogell, & Laurila, 2010), which may explain why eggshell spotting patterns are site‐specific. Although the repeatability of eggshell spottiness of individual females seems high (Skrade & Dinsmore, 2013; Wheelwright, Graff, & Norris, 2012), females may exhibit plasticity in eggshell coloration (i.e., in the quantities of pigments assigned to eggshells; e.g., Avilés, Stokke, Moksnes, Røskaft, & Møller, 2007; Wheelwright et al., 2012) depending on local factors, as this could optimize fitness in response to simultaneous changing threats (Hansson, 2004). Populations of African village weaverbirds *Ploceus cucullatus* changed in egg appearance after being introduced in sites where they experienced different levels of solar radiation compared to those in their original sites (Lahti, 2008). However, the variation described in these studies can also be interpreted either as local adaptation (evolution), or environmental constraints affecting egg coloration by modifying the state (nutritional status/stress level) of the female bird, and it remains to be demonstrated whether birds plastically adjust egg coloration to match environmental conditions, as it has been shown in insects (Abram et al., 2015; Torres‐Campos, Abram, Guerra‐Grenier, Boivin, & Brodeur, 2016). Individual phenotypic plasticity may be adaptive, as there may be differences in the effects of solar radiation throughout the long nesting season of the Kentish plover (individual females may lay up to four clutches in a season [Fraga & Amat, 1996]), as well as between sites at which individual plovers may breed during successive nesting attempts. Indeed, individual shorebirds may move thousands of kilometers to breed, not only between nesting seasons but also within seasons (Figuerola, 2007; Stenzel et al., 1994). In addition, under a climate‐warming scenario, the local adaptations may be altered, in which case phenotypic plasticity might be an important mechanism to respond to those changes. Therefore, historical and novel stressors of the environment may interact to shape spatial gradients in eggshell reflectance, and perhaps ultimately patterns of nest survival through effects on nest camouflage (e.g., Skrade & Dinsmore, 2013; Troscianko et al., 2016).

In conclusion, our study indicates that the latitudinal trends in eggshell coloration in the Kentish plover are affected by levels of solar radiation at the nesting sites, thus supporting the color‐mediated heating hypothesis. But solar radiation may affect the performance of embryos through a two‐way interaction between the risk of overheating and the deleterious effects of UV radiation. We have studied a single shorebird species, but different species may show different phenotypic responses to the same environmental gradient, meaning that differences between species in relative costs and benefits, especially due to differences in egg size, may affect latitudinal variations in eggshell color.

## CONFLICT OF INTEREST

The authors declare no conflict of interest.

## AUTHORS CONTRIBUTIONS

JAA and GLC conceived the idea. JAA, JG, CR, and MS designed the study. MS and JT contributed materials. JAA and CR collected the images. JG, GLC, CR, MAR, and JT analyzed the images. CR retrieved data of temperature and solar radiation from large databases. MAR ran the statistical analyses. JAA, JG, GLC, CR, and MAR wrote a first version, and all authors contributed to revisions of the manuscript.

## DATA ACCESSIBILITY

Data available from Digital.CSIC Repository: http://hdl.handle.net/10261/165880.
